# Stratified dosimetric comparison between high‐definition multileaf collimator‐based D2SRS and robotic radiosurgery in the treatment of brain metastases

**DOI:** 10.1002/acm2.70361

**Published:** 2025-11-29

**Authors:** Canyang Lin, Wei Liu, Nan Xiao, Yuliang Jiang, Junjie Wang

**Affiliations:** ^1^ Department of Oncology Longyan First Affiliated Hospital of Fujian Medical University Longyan Fujian China; ^2^ Department of Radiation Oncology Peking University Third Hospital Beijing China

**Keywords:** brain metastases, dosimetric comparison, high‐definition multileaf collimator, normal tissue paring, radiosurgery

## Abstract

**Purpose:**

To compare dosimetric characteristics and treatment efficiency between the high‐definition multileaf collimator (HD‐MLC) system and robotic radiosurgery (RRS) for brain metastases, with stratified analysis by lesion count.

**Methods:**

Twenty‐nine patients (12 single [SBM], 17 multiple [MBM] brain metastases) treated with RRS (CyberKnife) were re‐planned using a linear accelerator (LINAC) with 2.5‐mm micro‐MLC (D2SRS system). Key indices included target parameters (RTOG conformity index [RTOG CI], Paddick conformity index [Paddick CI], homogeneity index [HI], gradient index [GI], dose at 2 cm outside the target [D2cm]) and normal brain doses (V12Gy, V20Gy, V30Gy). The Wilcoxon signed‐rank test was used (*p* < 0.05).

**Results:**

In SBM, RTOG CI, Paddick CI, and GI showed no significant differences, but Paddick CI favored RRS for tumor volume (TV) < 80 cc and D2SRS for TV > 80 cc. D2SRS had better HI (0.08 ± 0.02 vs. 0.12 ± 0.03, *p* < 0.01); RRS had lower D2cm (18.2 ± 5.5 Gy vs. 23.8 ± 6.7 Gy, *p* = 0.008), V12Gy (5.3 ± 2.1 cc vs. 8.7 ± 3.5 cc, *p* < 0.01), and V20Gy (18.47 ± 21.17 cc vs. 35.30 ± 53.85 cc, *p* = 0.015), with similar V30Gy. D2SRS had shorter treatment time (TT) (8.2 ± 2.1 min vs. 26.8 ± 7.8 min, *p* < 0.01). In MBM, D2SRS outperformed RRS in RTOG CI (1.12 ± 0.21 vs. 1.45 ± 0.33, *p* = 0.007), Paddick CI (0.85 ± 0.11 vs. 0.68 ± 0.14, *p* = 0.007), and HI (0.07 ± 0.02 vs. 0.13 ± 0.03, *p* = 0.03). RRS had lower V12Gy (38.5 ± 12.6 cc vs. 48.1 ± 15.3 cc, *p* = 0.017), V20Gy (14.5 ± 4.2 cc vs. 21.3 ± 6.8 cc, *p* = 0.04), and V30Gy (14.99 ± 20.81 cc vs. 18.49 ± 23.19 cc, *p* = 0.026). D2SRS reduced TT by 77.3% (12.4 ± 3.5 min vs. 54.6 ± 15.2 min, *p* < 0.01). No significant differences in Dmax of the brainstem, optic nerves, or lenses were found.

**Conclusion:**

RRS provides better normal brain protection in both groups, while D2SRS shows superior conformity and efficiency in MBM. Clinical selection should depend on lesion burden.

## 1 BACKGROUND

Brain metastases pose a major challenge in the treatment of advanced cancer, affecting approximately 20%–40% of cancer patients, with their incidence continuing to rise alongside advancements in diagnostic imaging and systemic therapies.^1^ Fractionated stereotactic radiosurgery (fSRS) has emerged as a preferred alternative to single‐fraction stereotactic radiosurgery (SRS),^2^, ^3^, ^4^, ^5^, ^6^ as it achieves comparable tumor control rates through optimized normal tissue sparing while reducing the risk of radiation‐induced complications by 30%–50%.^7^, ^8^ Modern fSRS delivery platforms primarily include linear accelerators (LINACs) equipped with multileaf collimators (MLCs) and robotic radiosurgery systems (RRS, such as CyberKnife), each offering distinct dosimetric advantages.^9^, ^10^ Conventional LINACs have inherent geometric limitations (leaf width ≥5 mm), leading to reduced dose conformity for small targets (< 3 cm) (resulting in a 15%–20% decrease in the conformity index, CI) and suboptimal dose gradients near critical structures.^11^ In contrast, RRS platforms provide superior spatial precision (0.5–0.7 mm) and non‐isocentric beam delivery, achieving approximately a 30% improvement in the gradient index (GI) for solitary lesions.^12^ However, their fixed collimation systems require time‐consuming parameter adjustments when treating multiple metastases (adding 15–20 min per additional target), significantly reducing clinical treatment efficiency.^13^, ^14^

The development of high‐definition multileaf collimator (HD‐MLC) systems (with a leaf width of 2.5 mm), combined with Monte Carlo dose calculation and intelligent arc optimization, has successfully addressed the limitations of conventional LINACs.^15^ These systems reduce treatment time (TT) by more than 40% while maintaining excellent dosimetric performance.^16^, ^17^ Recent advancements in non‐coplanar arc technology have further enabled HD‐MLC platforms to achieve conformity and gradient metrics comparable to those of RRS.^18^ Despite these technological innovations, there remains a lack of comprehensive comparative studies between modern HD‐MLC and RRS platforms across varying metastatic burdens. Most previous studies have focused solely on solitary brain metastases (SBM) or failed to conduct stratified analyses based on lesion characteristics,^19^ leaving a critical research gap—the need to synchronously evaluate dosimetric performance, treatment efficiency, and normal brain tissue protection while stratifying patients by both lesion count and tumor volume (TV).^20^ To address this gap, there is an urgent need for research conducting stratified analyses based on lesion count (SBM: 1 lesion; multiple brain metastases [MBM]: 2–11 lesions) and TV.

The study hypothesizes that there are significant differences in dosimetric characteristics (including conformity, homogeneity, and gradient indices) and treatment efficiency between HD‐MLC‐based dynamic adaptive radiosurgery (D2SRS) and RRS in the treatment of brain metastases. The advantages of both systems are influenced by TV—RRS is more suitable for small‐volume tumors, while D2SRS is better suited for large‐volume tumors. Validating this hypothesis will provide evidence‐based guidance for optimizing the selection of clinical treatment platforms based on lesion burden.​

## 2 MATERIALS AND METHODS

### 2.1 Patient selection

This retrospective study enrolled 29 patients with brain metastases who received robotic radiosurgery (RRS; CyberKnife Model M5, Accuray Incorporated) in the Department of Radiation Oncology of our hospital. A total of 75 intracranial metastatic lesions were identified among these 29 patients, with a mean lesion volume of 24.14 cc (range: 0.83–163.40 cc). Among the patients, 12 had SBM, and 17 had MBM with 2–11 lesions per patient. The distribution of lesion counts and the proportion of patients in each subgroup are detailed in Table 1.

**TABLE 1:** Frequency distribution of the number of brain metastases lesions.

Number of lesions	Number of cases	Frequency (%)
1	12	41.4
2–5	9	31
6–10	5	17.2
11	3	10.3

The inclusion criteria for patients were as follows: (1) confirmation of brain metastases by pathological examination or imaging studies; (2) no prior history of radiotherapy to the brain; (3) good performance status, defined as an Eastern Cooperative Oncology Group (ECOG) performance status score of 0–2; (4) complete clinical and imaging data. Treatment regimens were individualized, with a prescribed dose of 21–40 Gy delivered in 3–5 fractions. This study was approved by the Institutional Ethics Committee of our hospital, and informed consent was obtained from all patients. All data used for analysis were anonymized.

### 2.2 CT scan acquisition parameters

All patients underwent brain CT scans using a 64‐slice spiral CT scanner (GE Discovery CT750 HD). The scan parameters were set as follows: tube voltage of 120 kV, tube current of 250 mA, slice thickness of 1.5 mm, slice interval of 0.5 mm, scan field of view (FOV) of 25 cm × 25 cm, and matrix size of 512 × 512. The standard reconstruction algorithm was adopted, and the scan range covered from the top of the skull to the base of the skull to ensure complete visualization of all brain metastatic lesions.

### 2.3 RRS (CyberKnife M5) treatment planning

CyberKnife treatments employ 6‐megavolt (MV) Flattening‐Filter‐Free (FFF) beams, delivering non‐coplanar irradiation via a robotic arm with a maximum dose rate of 1000 monitor units (MUs) per minute. Iris collimators (with apertures ranging from 5 to 60 mm) are selected based on lesion size, and skull tracking technology is used to ensure submillimeter positioning accuracy. The planning target volume (PTV) margin for CyberKnife is set at 0.5–1.0 mm. Treatment plans are created using MultiPlan software (Version 5.5.2, Accuray Incorporated, Sunnyvale, California, USA) and adopt a combined collimator strategy: a primary collimator matching the largest area of the lesion is used as the basis, paired with a smaller‐aperture collimator to optimize dose distribution at the lesion edges. Prescribed doses are determined according to the 70%–80% isodose line to balance target conformity and normal tissue protection needs. CyberKnife planning does not incorporate Monte Carlo dose calculation; instead, it uses a proprietary dose calculation algorithm optimized specifically for its non‐isocentric irradiation mode.

### 2.4 D2SRS (HD‐MLC system) planning

**FIGURE 1:**
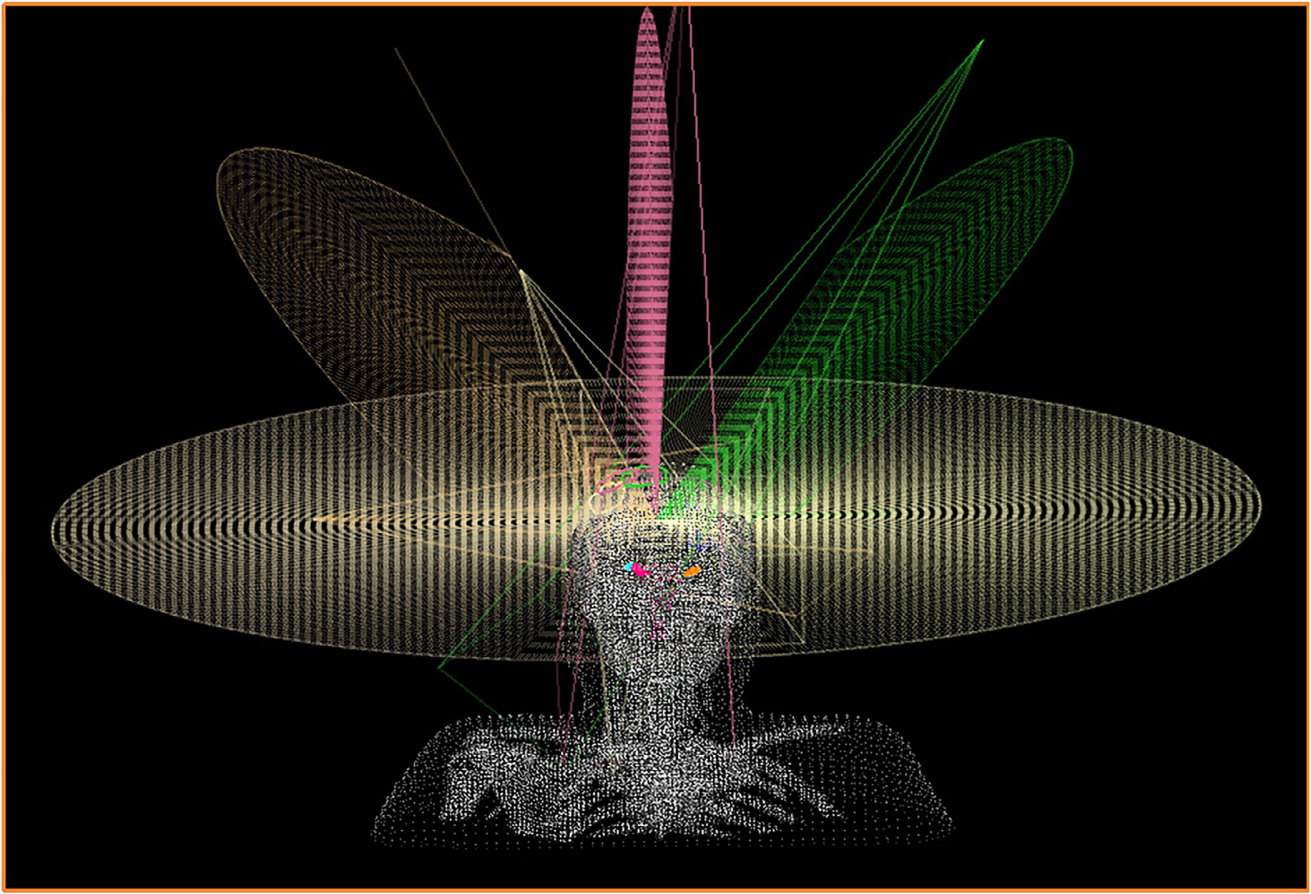
Schematic diagram showing four non‐coplanar arc angles (0°, 45°, 90°, 315°) for beam arrangement in treatment planning.

The HD‐MLC‐based D2SRS system (LinacTech), built on the VenusX LINAC platform (LinacTech, Beijing), features a 2.5 mm HD‐MLC (51 pairs, max field size 12 cm × 10 cm) and a Monte Carlo dose calculation algorithm, with a PTV margin (0.5–1 mm) consistent with RRS plans. Its treatment plan development involves four key steps: first, import DICOM images, PTV, and organ‐at‐risk (OAR) contour files, then define a 5 mm‐wide dose fall‐off ring (min 3 mm from PTV) and reference points—single‐lesion plans set isocenter at PTV's geometric center, while multiple‐lesion plans position it to “minimize the maximum distance to each PTV”; second, individualize prescription doses (consistent with original RRS dose when redeveloping) and adopt “one coplanar arc + three non‐coplanar arcs” (coplanar gantry angle 181°–179°, non‐coplanar corresponding to couch angles 45°/315°/90° and gantry angles 0°–179°/181°–0°/0°–181°), optimizing field angles/weights via TiGRT software (V2.0); third, conduct fluence optimization with dynamic segment modulation, followed by Monte Carlo dose calculation (grid size 3 mm × 3 mm × CT slice thickness) and three‐round iterative optimization (100 iterations/round, focusing on collimator angle adjustment); finally, adjust objective function weights to balance target CI, homogeneity index (HI), and normal tissue dose until meeting clinical standards (beam geometry shown in Figure 1).

### 2.5 Target dosimetric indicators

This study adopted an internationally recognized dosimetric evaluation system in the field of radiation oncology and systematically assessed the quality of treatment plans through the following key parameters:

**D95/D98**: Respectively represent the minimum dose received by 95% and 98% of the target volume, reflecting the dose coverage of the overall target and its edges.

**V95/V100**: Refer to the percentage of the target volume that receives at least 95% and 100% of the prescribed dose, respectively, and are used to evaluate the coverage range of the prescribed dose and the risk of hot spots.

**RTOG conformity index (RTOG CI)**: Calculated as RTOG CI = Vtarget,95%/(VPTV ∩ V95%), where Vtarget,95% is the target volume receiving 95% of the prescribed dose, VPTV is the total PTV, and V95% is the total volume receiving 95% of the prescribed dose. This index is used to quantify the degree of geometric matching between the 95% isodose surface and the target.

**Paddick conformity index (Paddick CI)**: Calculated as Paddick CI = (Vtarget,95%)^2^/(VPTV × V95%), which can comprehensively assess target coverage and normal tissue protection effects.

**Homogeneity index (HI)**: Calculated as HI = D5%/D95%, where D5% is the dose received by 5% of the target volume and D95% is the dose received by 95% of the target volume. It is used to characterize the uniformity of dose distribution within the target. For heterogeneous dose distributions, HI remains a feasible evaluation indicator, but its interpretation needs to be combined with clinical requirements. This study did not set a limit on the maximum dose, but required that it should not exceed 120% of the prescribed dose to avoid excessive damage to normal tissues caused by local high doses.

**GI**: Calculated as GI = V50%/V100%, where V50% is the volume receiving 50% of the prescribed dose and V100% is the volume receiving 100% of the prescribed dose. It reflects the steepness of dose fall‐off outside the target.

**Maximum dose at 2 cm outside the target (D2cm)**: Used to evaluate the radiation exposure risk of distant normal tissues.

All parameters were accurately calculated based on three‐dimensional dose distribution data, providing an objective basis for the optimization of treatment plans.

### 2.6 Indicators related to normal tissues, OARs, and treatment efficiency

#### 2.6.1 Indicators for normal tissues and OARs

**Normal brain tissue dose (V12Gy/V20Gy/V30Gy)**: Refers to the volume of normal brain tissue that receives a dose of ≥12 Gy, ≥20 Gy, and ≥30 Gy, respectively. A lower value indicates a lower risk of radiation‐induced damage.

**Organ‐at‐risk dose**: The dose constraints for OAR are set as follows: maximum dose (Dmax) to the brainstem ≤15 Gy, maximum dose (Dmax) to the optic nerve ≤10 Gy, and maximum dose (Dmax) to the lens ≤5 Gy.

#### 2.6.2 Treatment efficiency indicators

**Total MUs**: Represents the workload of the equipment; a lower value indicates a smaller operational burden.

**TT**: Refers to the duration of the irradiation operation; a shorter time indicates higher treatment efficiency.

### 2.7 Statistical methods

IBM SPSS Statistics 23.0 was used for statistical analyses, and GraphPad Prism 8 was employed for graphing and visualizing differences. The Wilcoxon signed‐rank test was used for comparisons, with a significance level of *p* < 0.05. Linear regression was applied to analyze correlations between key dosimetric indicators (CI, GI, and normal brain V12Gy) and TV. Ninety‐five percent confidence intervals (CIs) were added for all key parameters to provide a more comprehensive assessment of differences.

## 3 RESULTS

### 3.1 Visual characteristics of dose distribution

**FIGURE 2:**
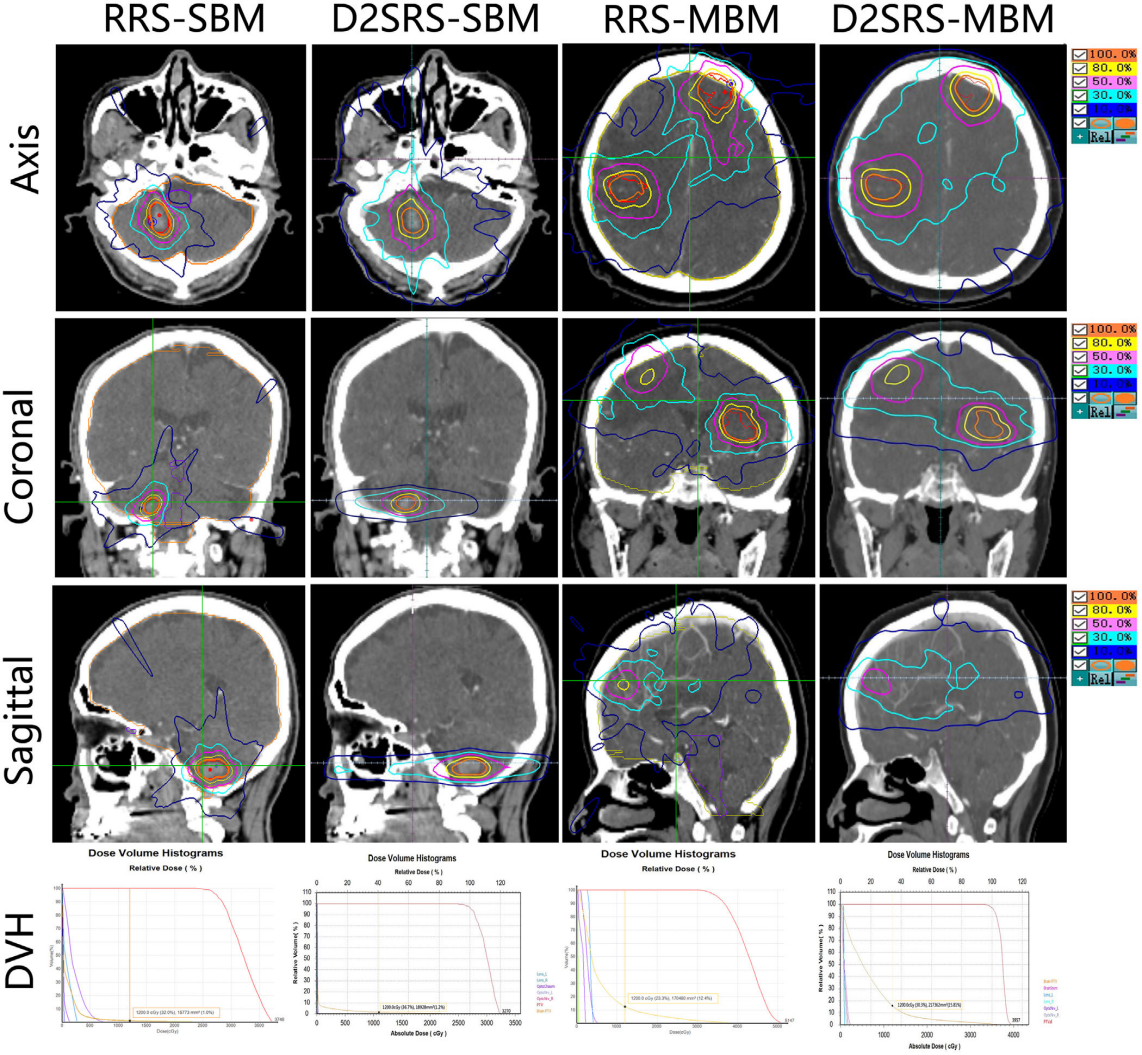
Shows the axial, coronal, and sagittal CT dose distributions of single‐brain metastasis (SBM) cases (RRS‐SBM group, D2SRS‐SBM group) and multiple‐brain metastasis (MBM) cases (RRS‐MBM group, D2SRS‐MBM group). Color‐coded contours correspond to the dose scale; the DVH of each group is labeled with V12Gy of normal brain tissue. D2SRS, DVH, dose‐volume histogram; RRS, robotic radiosurgery.

Figure 2 visually compares the dose distribution differences between D2SRS and RRS in cases of SBM and MBM through axial, coronal, and sagittal CT dose distribution maps, as well as dose‐volume histogram (DVH). Clear observations from the images are as follows: Compared with D2SRS, RRS demonstrates a more compact low‐dose distribution, which is specifically reflected in the smaller volumetric extent of the 10% and 30% isodose curves on dose distribution maps, corresponding to a reduced volume of normal brain tissue receiving ≥12 Gy (V12Gy) on DVH. Meanwhile, D2SRS exhibits superior target volume conformity, with improved geometric congruence between the high‐dose distribution and the target volume anatomical shape.

### 3.2 Comparison of target volume dose parameters

#### 3.2.1 Single brain metastasis (SBM) group

Data in Table 2 shows that there are significant differences in the target volume dose parameters between D2SRS and RRS in the SBM group: Compared with D2SRS, RRS has lower indicators related to target volume coverage (D95: 26.99 ± 2.44 Gy vs. 27.43 ± 2.61 Gy, *p* < 0.05; D98: 26.01 ± 2.41 Gy vs. 26.68 ± 2.56 Gy, *p* < 0.05), while the maximum dose within the target volume is significantly higher (36.72 ± 4.93 Gy vs. 32.95 ± 2.74 Gy, *p* = 0.015). In contrast, D2SRS exhibits more adequate target volume coverage capability (V95%: 99.44 ± 0.64% vs. 98.61 ± 0.70%, *p* = 0.012) and better dose uniformity (HI: 1.23 ± 0.07 vs. 1.36 ± 0.11, *p* = 0.015).

**TABLE 2:** Comparison of target dosimetric parameters between RRS and HD‐MLC‐based dynamic stereotactic radiosurgery (D2SRS) in patients with single (SBM) and multiple brain metastases (MBM).

Parameters	RRS (mean ± SD)—SBM	D2SRS (mean ± SD)—SBM	*p*‐value	95% CI	RRS (mean ± SD)—MBM	D2SRS (mean ± SD)—MBM	*p*‐value	95% CI
D95 (Gy)	26.99 ± 2.44	27.43 ± 2.61	**0.002**	**[−0.78, −0.10]**	28.96 ± 5.34	28.80 ± 5.14	0.408	[−1.32, 1.00]
D98 (Gy)	26.01 ± 2.41	26.68 ± 2.56	**0.005**	**[−0.97, −0.37]**	27.65 ± 5.29	27.90 ± 5.11	0.215	[−0.83, 0.33]
Max Dose (Gy)	36.72 ± 4.93	32.95 ± 2.74	**0.015**	**[1.23, 6.31]**	39.70 ± 7.12	35.89 ± 6.49	**0.007**	**[1.21, 6.41]**
Min Dose (Gy)	23.36 ± 2.40	24.52 ± 2.98	**0.041**	**[−2.02, −0.10]**	23.40 ± 5.07	24.52 ± 7.87	**0.031**	**[−2.20, −0.04]**
V95%	98.61 ± 0.70	99.44 ± 0.64	**0.012**	**[0.23, 1.43]**	98.69 ± 0.90	99.21 ± 0.80	0.076	[−0.03, 1.07]
V100%	96.64 ± 2.03	96.61 ± 1.95	0.388	[−0.85, 0.79]	96.02 ± 0.90	94.92 ± 4.13	0.795	[−2.18, 4.38]
Paddick CI	0.77 ± 0.08	0.74 ± 0.15	0.583	[−0.07, 0.13]	0.68 ± 0.09	0.75 ± 0.09	**0.01**	**[0.02, 0.12]**
RTOG CI	1.29 ± 0.19	1.38 ± 0.39	0.53	[−0.18, 0.36]	1.42 ± 0.18	1.28 ± 0.17	**0.009**	**[−0.22, −0.06]**
HI	1.36 ± 0.11	1.23 ± 0.07	**0.015**	**[−0.19, −0.03]**	1.39 ± 0.10	1.26 ± 0.13	**0.009**	**[−0.20, −0.06]**
GI	3.78 ± 1.10	3.74 ± 0.74	0.875	[−0.52, 0.60]	5.43 ± 4.01	5.61 ± 4.03	0.163	[−0.65, 0.29]
D2cm (Gy)	9.89 ± 3.57	15.06 ± 5.29	**0.002**	**[−7.85, −2.59]**	14.90 ± 5.24	14.91 ± 3.25	0.586	[−1.87, 1.89]

*Note*: D95/D98 refers to the minimum dose to 95%/98% of the PTV, and Max/Min Dose represents the dose range within the PTV; V95%/V100% denotes the percentage of the PTV that receives ≥95%/100% of the prescribed dose; Paddick CI is calculated as (Vtarget,95%)^2^/(VPTV × V95%), and RTOG CI is calculated as Vtarget,95%/(VPTV∩V95%); HI is calculated as HI = D5%/D95%, and GI is calculated as GI = V50%/V100%; D2cm is the maximum dose at 2 cm outside the PTV. Statistically, *p* < 0.05 indicates a statistically significant difference; bold values in the table represent the superior performance of the corresponding parameters.

Abbreviations: D2cm, dose at 2 cm outside the target; GI, gradient index; HI, homogeneity index; Paddick CI, Paddick conformity index; PTV, planning target volume; RRS, robotic radiosurgery.

**FIGURE 3:**
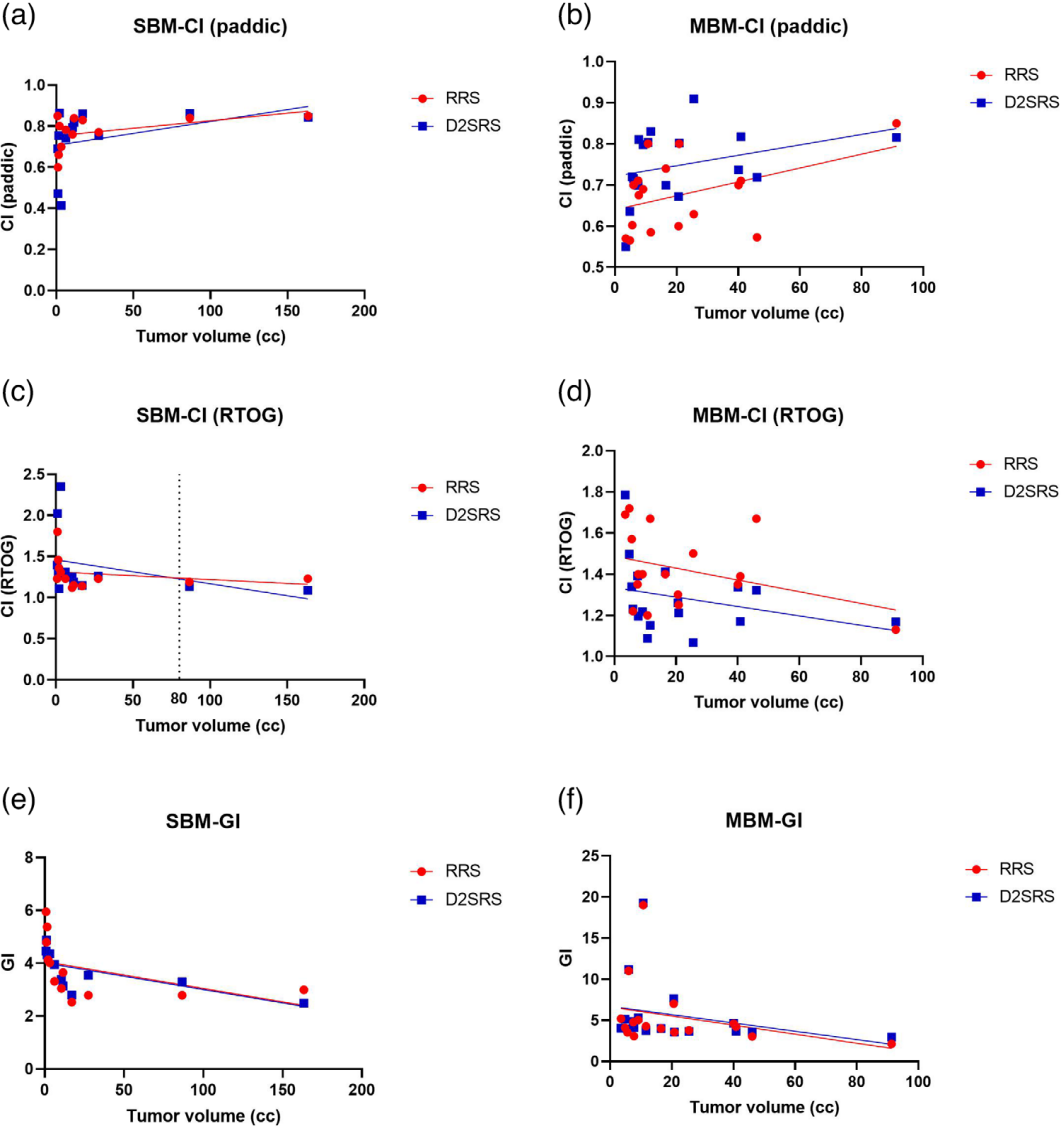
Panels (a)–(f) are scatter plots of CI and GI changing with TV in single/multiple brain metastases (SBM/MBM). The red color represents RRS, and the blue color represents D2SRS, presenting the dose conformity and gradient performances of the two techniques under different tumor burdens. CI, conformity index; GI, gradient index; RRS, robotic radiosurgery.

Further stratified analysis by TV (Figure 3) reveals that the conformity performance of the two techniques is associated with TV: When TV < 80 cc, the RTOG CI of RRS is superior; when TV > 80 cc, the Paddick CI of D2SRS surpasses that of RRS and shows better performance. The GI has no obvious fluctuation under different TVs and remains stable. Overall, there are no statistically significant differences in conformity indices (RTOG CI, Paddick CI) and GI between the two groups.

#### 3.2.2 Multiple brain metastases (MBM) group

The target volume dose parameters of the MBM group show different characteristics from those of the SBM group (Table 2): The maximum dose within the target volume of RRS is still significantly higher than that of D2SRS (39.70 ± 7.12 Gy vs. 35.89 ± 6.49 Gy, *p* = 0.007), but there are no significant differences in the core indicators of target volume coverage (D95, D98, V95%, V100%) between the two groups. Notably, D2SRS has more prominent advantages in conformity and uniformity: In terms of conformity indices, both the RTOG CI (1.28 ± 0.17 vs. 1.42 ± 0.18, *p* = 0.009) and Paddick CI (0.75 ± 0.09 vs. 0.68 ± 0.09, *p* = 0.010) of D2SRS are significantly superior; the HI is significantly lower (1.26 ± 0.13 vs. 1.39 ± 0.10, *p* = 0.009), while there is no significant difference in GI.

Stratified analysis by the number of lesions (Table 3) shows that as the number of lesions increases (2–5 lesions, 6–10 lesions, 11 lesions), the advantage of D2SRS in conformity (CI) gradually enhances. Moreover, under different TVs, D2SRS always maintains a high CI value and stable GI (Figure 3), suggesting that it has better stability in dose conformity in complex scenarios with multiple lesions.

**TABLE 3:** Comparison of RTOG CI among different lesion count subgroups in the MBM cohort.

Lesion count subgroup (*n*)	Treatment modality	RTOG CI (mean ± SD)	Magnitude of difference versus RRS (%)	*p*‐value	95% CI
2–5	RRS	1.43 ± 0.17	–	–	–
	D2SRS	1.32 ± 0.15	7.7	**0.089**	**[−0.25, 0.03]**
6–10	RRS	1.45 ± 0.19	–	–	–
	D2SRS	1.21 ± 0.11	16.5	**0.023**	**[−0.41, −0.07]**
11	RRS	1.48 ± 0.20	–	–	–
	D2SRS	1.10 ± 0.08	25.7	**0.006**	**[−0.55, −0.21]**

*Note*: An RTOG CI value closer to 1 indicates better dose conformity. The magnitude of difference is calculated as [(RTOG CI of RRS—RTOG CI of D2SRS)/RTOG CI of RRS] × 100%. A positive value means D2SRS is superior to RRS, and a larger value indicates a more significant advantage.

Abbreviations: RRS, robotic radiosurgery; RTOG, RTOG conformity index.

### 3.3 Protective effects on normal brain tissue and OARs

#### 3.3.1 Single brain metastasis (SBM) group

Data in Table 4 indicates that RRS shows better performance in protecting normal brain tissue in the SBM group: The volume of normal brain tissue receiving doses of ≥12 Gy (V12Gy: 38.76 ± 47.55 cc vs. 66.44 ± 98.43 cc, *p* = 0.019) and ≥20 Gy (V20Gy: 18.47 ± 21.17 cc vs. 35.30 ± 53.85 cc, *p* = 0.015) is reduced by 39.1% and 47.7%, respectively, compared with D2SRS. D2cm is also reduced by 34.3% (9.89 ± 3.57 Gy vs. 15.06 ± 5.29 Gy, *p* = 0.002). Only the difference in the volume of normal brain tissue receiving a dose of ≥30 Gy (V30Gy) does not reach statistical significance.

**TABLE 4:** Comparison of normal tissue/OAR doses and treatment efficiency between RRS and D2SRS in patients with single (SBM) and multiple brain metastases (MBM).

Parameters	RRS (mean ± SD)—SBM	D2SRS (mean ± SD)—SBM	*p*‐value	95% CI	RRS (mean ± SD)—MBM	D2SRS (mean ± SD)—MBM	*p*‐value	95% CI
Brain stem (Dmax)	4.20 ± 4.37	3.03 ± 3.78	**0.019**	**[0.23, 2.11]**	8.92 ± 6.76	8.06 ± 7.67	0.227	[−1.58, 3.34]
Optic nerve (Dmax)	1.12 ± 0.96	0.11 ± 0.09	**0.075**	**[**−**0.02, 2.04]**	2.47 ± 3.64	0.85 ± 0.63	0.3	[−1.16, 4.60]
Lens (Dmax)	0.28 ± 0.48	0.13 ± 0.14	1	[−0.23, 0.53]	0.37 ± 0.34	0.61 ± 0.33	0.152	[−0.08, 0.54]
V12Gy (cc)	38.76 ± 47.55	66.44 ± 98.43	**0.019**	**[**−**45.36,** −**5.90]**	123.89 ± 98.54	176.91 ± 138.36	**<0.001**	**[**−**82.74,** −**23.30]**
V20Gy (cc)	18.47 ± 21.17	35.30 ± 53.85	**0.015**	**[**−**29.51,** −**3.15]**	46.47 ± 40.93	62.42 ± 42.00	**0.001**	**[**−**25.67,** −**6.23]**
V30Gy (cc)	3.10 ± 5.91	5.89 ± 8.78	0.066	[−5.67, 0.09]	14.99 ± 20.81	18.49 ± 23.19	**0.026**	**[**−**6.42,** −**0.58]**
MU	20472 ± 9228	4165 ± 913	**0.002**	**[**−**20265.38,** −**12348.62]**	37903 ± 13719	5191 ± 1958	**<0.001**	**[**−**37674.52,** −**27550.48]**
TIME (min)	28.92 ± 5.23	9.60 ± 5.09	**0.003**	**[**−**24.50,** −**5.14]**	37.35 ± 7.36	9.58 ± 2.54	**<0.001**	**[**−**33.69, ‐21.85]**

*Note*: Dmax refers to the maximum dose received by OAR; V12Gy/V20Gy/V30Gy denotes the volume of normal brain tissue that receives a dose of ≥12 Gy/20 Gy/30 Gy. Statistically, *p* < 0.05 indicates a statistically significant difference; bold values in the table represent the superior performance of the corresponding parameters.

Abbreviations: MU, monitor unit; OAR, organ‐at‐risk; RRS, robotic radiosurgery.

In terms of doses to OAR, the maximum dose to the brainstem in RRS is higher (4.20 ± 4.37 Gy vs. 3.03 ± 3.78 Gy, *p* = 0.019), but there are no significant differences in the doses to the optic nerve and lens between the two groups, and all doses meet clinical safety thresholds.

#### 3.3.2 Multiple brain metastases (MBM) group

**FIGURE 4:**
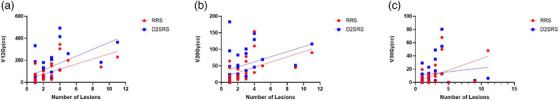
Panels (a), (b), and (c) are scatter plots of V12Gy, V20Gy, and V30Gy changing with the number of lesions, respectively. The red color represents RRS, and the blue color represents D2SRS, showing the dose‐volume situations of the two techniques under different numbers of lesions. RRS, robotic radiosurgery.

In the MBM group, the advantage of RRS in protecting normal brain tissue remains significant, as detailed in Table 4: V12Gy (123.89 ± 98.54 cc vs. 176.91 ± 138.36 cc, *p* < 0.001), V20Gy (46.47 ± 40.93 cc vs. 62.42 ± 42.00 cc, *p* = 0.001), and V30Gy (14.99 ± 20.81 cc vs. 18.49 ± 23.19 cc, *p* = 0.026) were all significantly lower than those achieved with D2SRS. This protective advantage remained consistent across varying numbers of lesions, further supported by the data trends shown in Figure 4.

As further summarized in Table 4, considerable variability was observed in V12Gy and V20Gy values within the MBM cohort—similar to the SBM group—which is likely attributable to the complex spatial distribution of multiple metastases and substantial dose interference between adjacent lesions. Despite these variations, Table 4 also demonstrates that no significant differences were detected in maximum doses to critical neural structures, including the brainstem, optic nerves, and lenses, between the two techniques. All recorded OAR doses fell within established clinical safety thresholds.

### 3.4 Comparison of treatment efficiency

As shown in Table 4, D2SRS demonstrated significantly superior treatment efficiency compared to RRS in both the SBM and MBM groups: In both cohorts, the MU and irradiation time required for D2SRS were substantially lower than those for RRS, with statistically significant differences (*p* < 0.01). These results indicate that D2SRS can effectively reduce treatment operational costs and enhance clinical workflow efficiency across varying brain metastasis burdens.

## 4 DISCUSSION

This study systematically analyzed the dosimetric characteristics of 29 patients with brain metastases, elucidating the technical features of the HD‐MLC‐based D2SRS LINAC system and the RRS. For the first time, the differential clinical advantages of these two techniques under varying tumor burdens were revealed. In SBM, RRS demonstrated superior protection of normal brain tissue compared to D2SRS, with V12Gy reduced by 39.1% (*p* < 0.01) and D2cm reduced by 34.3% (*p* = 0.002), reflecting a steeper dose gradient. When the TV was < 80 cc, RRS showed better conformity than D2SRS, whereas the opposite was true for TV > 80 cc. Leveraging the dynamic shaping capability of the 2.5 mm HD‐MLC, D2SRS achieved higher conformity (RTOG CI improved by 10.9%, *p* = 0.009) and treatment efficiency (time reduced by 77.3%, *p* < 0.001) in MBM. In addition, the maximum doses to OARs showed no significant differences between the two techniques, both meeting clinical safety requirements.

In SBM treatment, the RRS group exhibited slightly lower D95/D98 coverage and poorer dose homogeneity (HI: 1.36 ± 0.11 vs. 1.23 ± 0.07, *p* = 0.015). This phenomenon primarily stems from the technical characteristics of RRS: its non‐isocentric irradiation mode combined with circular collimators creates a “hotspot‐focused” high‐dose concentration zone.^9^ While this technique achieves rapid dose fall‐off at the tumor periphery, it also leads to more pronounced dose hotspots within the target, thereby compromising overall dose homogeneity.^13^ However, RRS delivered significantly lower maximum D2cm and effectively reduced the volume of normal brain tissue exposed to low‐dose radiation (lower V12Gy).^14^ In contrast, D2SRS, employing multi‐arc dispersed irradiation and HD‐MLC dynamic shaping, resulted in slightly lower brainstem doses.^10^, ^19^ Although RRS delivers moderately higher doses to the brainstem near the target due to its technical design, the doses from both techniques remain well below clinical safety thresholds.^20^ Furthermore, the steep dose gradient of RRS effectively controls low‐dose irradiation to the whole brain, complementing D2SRS and offering differentiated clinical options, particularly for cases requiring stringent protection of peritumoral normal tissue.^6^

In the treatment of brain metastases, several studies have compared CyberKnife (an RRS system) with LINAC‐based techniques. For instance, both Zhang et al.^13^ and Thomas et al.^14^ reported that LINAC‐based techniques offer advantages in treatment efficiency, though with slightly inferior normal tissue protection—consistent with the findings of this study. However, these studies, along with those by Yamamoto^5^ and Calugaru,^6^ did not perform stratified analyses based on TV. This study found that although there was no significant difference in overall CI between RRS and D2SRS, Paddick CI exhibited a clear volume dependence: for tumors < 80 cc, RRS showed approximately 5.3% better Paddick CI than D2SRS, owing to the superior geometric match of its circular collimators with small target volumes; for tumors > 80 cc, the 2.5 mm HD‐MLC multi‐arc dynamic shaping technology of D2SRS demonstrated superior conformity. This finding aligns with Ruggero et al.^21^ observation regarding the advantage of MLC systems for larger lesions. Our Figure 1 visually confirms this phenomenon. These results suggest that clinical technique selection should fully consider TV to achieve optimal target coverage, and future studies with finer volume stratification are needed to further clarify these dosimetric differences.

Consistent with studies by Hartgerink et al.,^3^ this study found that for MBM, D2SRS significantly outperformed RRS in RTOG CI, Paddick CI, and HI, primarily attributable to the precise dynamic shaping capability of the 2.5 mm HD‐MLC and the high accuracy of the Monte Carlo dose calculation algorithm. It should be noted that the HD‐MLC technology used here is not conventional VMAT but an advanced shaping technology based on it, achieving superior dose modulation and conformity through smaller leaves and more flexible arc optimization. Although the two techniques showed comparable GI, indicating similar dose fall‐off characteristics between D2SRS's multi‐arc rotational dose superposition and RRS's non‐isocentric decay, important differences emerged in normal tissue protection: during multi‐target treatments, frequent field adjustments in RRS easily lead to interference and superposition of dose gradients,^12^ necessitating larger arc separation angles that increase planning complexity and time; whereas the single‐isocenter multi‐arc technique of D2SRS, despite causing larger low‐dose exposure volumes (e.g., V12Gy, V20Gy) in normal brain tissue due to longer beam paths,^18^ achieves better target conformity and homogeneity. This reveals a complementary relationship: D2SRS is more suitable for MBM scenarios prioritizing target coverage and treatment efficiency, while RRS retains advantages in protecting normal tissue from low‐dose irradiation.

This study shows D2SRS has notable efficiency advantages in brain metastasis treatment: its single‐isocenter multi‐arc technique simplifies workflow and shortens TT, with particularly prominent benefits in cases of high tumor burden.^22^ Despite broader low‐dose spread (e.g., higher V12Gy), D2SRS’ balance between conformity and efficiency enables same‐day or outpatient treatment, critical for patients with poor performance status or needing concurrent systemic therapy.^23^ In recent years, LINAC‐based SRS technologies have continued to evolve. Novel techniques represented by Varian's HyperArc, which also employs single‐isocenter irradiation for multiple targets, have been proven to offer higher efficiency and better dosimetric performance than conventional VMAT;^24^ research by Jung et al.^16^ also showed that for MBM with more than 20 lesions, HyperArc maintains high treatment efficiency while delivering improved conformity and homogeneity. Although this study did not directly compare D2SRS with HyperArc, the high similarity in their technical principles (single‐isocenter multi‐arc combined with HD‐MLC) suggests that D2SRS also holds broad application prospects for treating MBM with numerous lesions. Future studies should further compare these two techniques to provide more individualized options for clinical practice.

This study has several limitations. First, it only focuses on dosimetric comparisons and does not assess long‐term clinical outcomes, making it unclear whether the relevant dosimetric advantages can be translated into tangible clinical benefits, which requires verification through prospective follow‐up studies. Second, all patients initially received RRS treatment, while D2SRS plans were designed retrospectively in a simulated environment—there are differences in optimization conditions between the two, and potential subjective biases in the replanning process may affect the results. Finally, as a single‐center retrospective study with a small sample size, the generalizability of the research conclusions is limited; considering the differences in patient characteristics and treatment protocols across different centers, further verification of the conclusions through multi‐center, large‐sample studies is needed.

## 5 CONCLUSION

Dosimetrically, RRS provides better normal brain tissue protection (lower V12Gy, V20Gy, and D2cm) in both SBM and MBM groups, with superior conformity for SBM < 80 cc. By contrast, D2SRS exhibits superior conformity (higher RTOG CI and Paddick CI) and homogeneity (lower HI) for SBM > 80 cc and all MBM cases, along with significant advantages in treatment efficiency (lower MU and shorter time)—especially for MBM. Clinically, treatment selection should align with lesion burden: RRS is preferred for small‐volume SBM (< 80 cc) requiring strict normal tissue protection (e.g., lesions near eloquent regions), while D2SRS is suitable for large‐volume SBM (> 80 cc) and MBM, particularly when efficiency is a key consideration (e.g., patients needing multiple treatments or with poor performance status). Notably, this study only provides dosimetric evidence—final decisions should also take into account patient condition, lesion location, and equipment availability—and future research needs to integrate clinical outcome data to refine treatment selection strategies.

## AUTHOR CONTRIBUTIONS

Canyang Lin, Yuliang Jiang, Wei Liu, and Nan Xiao collected and curated the data. Canyang Lin. performed formal analysis. Canyang Lin and Junjie Wang drafted the original manuscript. All authors critically reviewed and approved the final manuscript.

## CONFLICT OF INTEREST STATEMENT

The authors declare no conflicts of interest.

## ETHICS APPROVAL

This study was approved by the **Institutional Review Board of Peking University Third Hospital**. All procedures complied with the ethical principles of the **1964 Helsinki Declaration** and its subsequent amendments.

## Data Availability

All datasets generated and analyzed during this study are available within the published article and its supplementary materials.

## References

[1] Nayak L , Lee EQ , Wen PY . Epidemiology of brain metastases. Curr Oncol Rep. 2012;14(1):48‐54. doi:10.1007/s11912‐011‐0203‐y 22012633 10.1007/s11912-011-0203-y

[2] Gondi V , Bauman G , Bradfield L , et al. Radiation therapy for brain metastases: an ASTRO clinical practice guideline. Pract Radiat Oncol. 2022;12(4):265‐282. doi:10.1016/j.prro.2022.02.003 35534352 10.1016/j.prro.2022.02.003

[3] Hartgerink D , Swinnen A , Roberge D , et al. LINAC based stereotactic radiosurgery for multiple brain metastases: guidance for clinical implementation. Acta Oncol. 2019;58(9):1275‐1282. doi:10.1080/0284186x.2019.1633016 31257960 10.1080/0284186X.2019.1633016

[4] Brown PD , Jaeckle K , Ballman KV , et al. Effect of radiosurgery alone vs radiosurgery with whole brain radiation therapy on cognitive function in patients with 1 to 3 brain metastases: a randomized clinical trial. JAMA. 2016;316(4):401‐409. doi:10.1001/jama.2016.9839 27458945 10.1001/jama.2016.9839 PMC5313044

[5] Yamamoto M , Serizawa T , Shuto T , et al. Stereotactic radiosurgery for patients with multiple brain metastases (JLGK0901): a multi‐institutional prospective observational study. Lancet Oncol. 2014;15(4):387‐395. doi:10.1016/s1470‐2045(14)70061‐0 24621620 10.1016/S1470-2045(14)70061-0

[6] Calugaru E , Whiting Z , Delacruz B , et al. Direct dosimetric comparison of linear accelerator vs. Gamma Knife fractionated stereotactic radiotherapy (fSRT) of large brain tumors. Med Dosim. 2023;48(1):31‐36. doi:10.1016/j.meddos.2022.09.006 36503990 10.1016/j.meddos.2022.09.006

[7] Zachary AK , Yoshiya Y , Timothy AC , et al. Long‐term risk of radionecrosis and imaging changes after stereotactic radiosurgery for brain metastases. J Neurooncol. 2015;125(1):149‐156. doi:10.1007/s11060‐015‐1881‐3 26307446 10.1007/s11060-015-1881-3 PMC4726630

[8] Milano MT , Grimm J , Niemierko A , et al. Single‐ and multifraction stereotactic radiosurgery dose/volume tolerances of the brain. Int J Radiat Oncol Biol Phys. 2021;110(1):68‐86. doi:10.1016/j.ijrobp.2020.08.013 32921513 10.1016/j.ijrobp.2020.08.013 PMC9387178

[9] Chambrelant I , Jarnet D , Bou‐Gharios J , et al. Stereotactic radiation therapy of single brain metastases: a literature review of dosimetric studies. Cancers. 2023;15(15):3937. doi:10.3390/cancers15153937 37568753 10.3390/cancers15153937 PMC10416831

[10] Nikhil TS , Chase G , Ryan H , et al. Linear accelerator‐based radiosurgery is associated with lower incidence of radionecrosis compared with Gamma Knife for treatment of multiple brain metastases. Radiother Oncol. 2020;147(0):136‐143. doi:10.1016/j.radonc.2020.03.024 32294607 10.1016/j.radonc.2020.03.024

[11] Jin JY , Yin FF , Ryu S , Ajlouni M , Kim JH . Dosimetric study using different leaf‐width MLCs for treatment planning of dynamic conformal arcs and intensity‐modulated radiosurgery. Med Phys. 2005;32(2):405‐411. doi:10.1118/1.1842911 15789586 10.1118/1.1842911

[12] Ruggieri R , Naccarato S , Mazzola R , et al. Linac‐based VMAT radiosurgery for multiple brain lesions: comparison between a conventional multi‐isocenter approach and a new dedicated mono‐isocenter technique. Radiat Oncol. 2018;13(1):38. doi:10.1186/s13014‐018‐0985‐2 29506539 10.1186/s13014-018-0985-2 PMC5836328

[13] Zhang S , Yang R , Shi C , et al. Noncoplanar VMAT for brain metastases: a plan quality and delivery efficiency comparison with coplanar VMAT, IMRT, and CyberKnife. Technol Cancer Res Treat. 2019;18. doi:10.1177/1533033819871621 10.1177/1533033819871621 PMC6710677 31451059

[14] Thomas EM , Popple RA , Wu X , et al. Comparison of plan quality and delivery time between volumetric arc therapy (RapidArc) and Gamma Knife radiosurgery for multiple cranial metastases. Neurosurgery. 2014;75(4):409‐417. doi:10.1227/neu.0000000000000448 24871143 10.1227/NEU.0000000000000448 PMC4203364

[15] Q Jackie W , Zhiheng W , John PK , et al. Impact of collimator leaf width and treatment technique on stereotactic radiosurgery and radiotherapy plans for intra‐ and extracranial lesions. Radiat Oncol. 2009;4(0). doi:10.1186/1748‐717x‐4‐3 10.1186/1748-717X-4-3 PMC2637285 19159471

[16] Jung H , Yoon J , Dona Lemus O , et al. Dosimetric evaluation of LINAC‐based single‐isocenter multi‐target multi‐fraction stereotactic radiosurgery with more than 20 targets: comparing MME, HyperArc, and RapidArc. Radiat Oncol. 2024;19(1):19. doi:10.1186/s13014‐024‐02416‐7 38326813 10.1186/s13014-024-02416-7 PMC10848506

[17] Tanyi JA , Summers PA , McCracken CL , Chen Y , Ku LC , Fuss M . Implications of a high‐definition multileaf collimator (HD‐MLC) on treatment planning techniques for stereotactic body radiation therapy (SBRT): a planning study. Radiat Oncol. 2009;4:22. doi:10.1186/1748‐717x‐4‐22 19591687 10.1186/1748-717X-4-22 PMC2716348

[18] Clark GM , Popple RA , Prendergast BM , et al. Plan quality and treatment planning technique for single isocenter cranial radiosurgery with volumetric modulated arc therapy. Pract Radiat Oncol. 2012;2(4):306‐313. doi:10.1016/j.prro.2011.12.003 24674169 10.1016/j.prro.2011.12.003

[19] Zhu L , Dong S , Sun L , et al. Dosimetric comparison of HyperArc and InCise MLC‐based CyberKnife plans in treating single and multiple brain metastases. J Appl Clin Med Phys. 2024;25(8):e14404. doi:10.1002/acm2.14404 38803034 10.1002/acm2.14404 PMC11302820

[20] Redmond KJ , Robertson S , Lo SS , et al. Consensus contouring guidelines for postoperative stereotactic body radiation therapy for metastatic solid tumor malignancies to the spine. Int J Radiat Oncol Biol Phys. 2017;97(1):64‐74. doi:10.1016/j.ijrobp.2016.09.014 27843035 10.1016/j.ijrobp.2016.09.014 PMC5600487

[21] Ruggero R , Stefania N , Rosario M , et al. Linac‐based radiosurgery for multiple brain metastases: comparison between two mono‐isocenter techniques with multiple non‐coplanar arcs. Radiother Oncol. 2019;132(0):70‐78. doi:10.1016/j.radonc.2018.11.014 30825972 10.1016/j.radonc.2018.11.014

[22] Ciérvide R , Martí J , López M , et al. Single and multitarget stereotactic radiosurgery (SRS) with single isocenter in the treatment of multiple brain metastases (BM): institutional experience. Clin Transl Oncol. 2025;27(7):3183‐3197. doi:10.1007/s12094‐024‐03844‐3 39814975 10.1007/s12094-024-03844-3

[23] Wang J , Zheng Q , Wang Y , et al. Dosimetric comparison of ZAP‐X, Gamma Knife, and CyberKnife stereotactic radiosurgery for single brain metastasis. BMC Cancer. 2024;24(1):936. doi:10.1186/s12885‐024‐12710‐y 39090564 10.1186/s12885-024-12710-y PMC11295608

[24] Clark GM , Popple RA , Young PE , Fiveash JB . Feasibility of single‐isocenter volumetric modulated arc radiosurgery for treatment of multiple brain metastases. Int J Radiat Oncol Biol Phys. 2010;76(1):296‐302. doi:10.1016/j.ijrobp.2009.05.029 19836151 10.1016/j.ijrobp.2009.05.029

